# A Neural Network Approach to Smarter Sensor Networks for Water Quality Monitoring

**DOI:** 10.3390/s120404605

**Published:** 2012-04-10

**Authors:** Edel O'Connor, Alan F. Smeaton, Noel E. O'Connor, Fiona Regan

**Affiliations:** 1 CLARITY: Centre for Sensor Web Technologies, Dublin City University, Glasnevin, Dublin 9, Ireland; E-Mails: alan.smeaton@dcu.ie (A.F.S.); noel.oconnor@dcu.ie (N.E.O.); 2 MESTECH: Marine and Environmental Sensing Technology Hub, Dublin City University, Glasnevin, Dublin 9, Ireland; E-Mail: fiona.regan@dcu.ie

**Keywords:** multi-modal sensor networks, rainfall radar, chemical sensors, environmental monitoring, visual sensing

## Abstract

Environmental monitoring is evolving towards large-scale and low-cost sensor networks operating reliability and autonomously over extended periods of time. Sophisticated analytical instrumentation such as chemo-bio sensors present inherent limitations because of the number of samples that they can take. In order to maximize their deployment lifetime, we propose the coordination of multiple heterogeneous information sources. We use rainfall radar images and information from a water depth sensor as input to a neural network (NN) to dictate the sampling frequency of a phosphate analyzer at the River Lee in Cork, Ireland. This approach shows varied performance for different times of the year but overall produces output that is very satisfactory for the application context in question. Our study demonstrates that even with limited training data, a system for controlling the sampling rate of the nutrient sensor can be set up and can improve the efficiency of the more sophisticated nodes of the sensor network.

## 1. Introduction

The need to continuously protect, regulate and monitor the quality of water in both our coastal and freshwater environments is being recognised with the introduction of a growing body of legislation such as the EU Water Framework Directive (http://ec.europa.eu/environment/water/water-framework/info/intro_en.htm) issued in 2000. In these environments, an array of biological, chemical, geological and physical processes occur over a range of temporal and spatial scales. They are dynamic environments affected by a range of anthropogenic factors as well as naturally occurring processes.

For many years those responsible for managing our water resources relied solely on field measurements for coastal monitoring and water quality assessment. This involves costly, time and labour-intensive on-site sampling and data collection, transportation to laboratories for analysis, and then subsequent evaluation. This type of sampling is too limited in both temporal and spatial terms to adequately monitor the quality of water bodies on a long term basis, to model and understand key environmental processes, or to capture dynamic marine events which may pose a threat to the environment or human health. In the past this type of sampling has also introduced various data quality issues through inadequate quality-control and quality assurance protocols such as extended holding times before analysis and the use of non-standardised methodologies [1].

New technologies are emerging in order to enable remote autonomous sensing of our water systems and subsequently meet the demands for high temporal and spatial monitoring. In particular, advances in communication and sensor technology have provided a catalyst for progress in remote monitoring of our water systems [2]. In recent years the concept of wireless sensor networks (WSNs) has been the focus of much research. The concept is relatively new and involves a diverse range of technologies and disciplines all brought together into one cooperating system. In parallel, the demand for continuous assessment of nutrient concentrations in coastal and inland waters has also led to the development of novel analytical instruments using newly emerging technologies [3]. Combining these new sensing instruments with the concept of WSNs provides an opportunity for long-term data collection at scales and resolutions that are difficult or impossible to obtain otherwise. The data collection process is streamlined with a minimisation of human errors and time delays increasing the quantity, and quality of data on temporal and spatial scales with a possibility of real-time alert notifications of harmful marine events [1]. Data can also be accessed remotely, which removes the need for data collection in sometimes hazardous or hard to reach environments.

However, despite continuous improvements there are still limitations with the use of this technology in environmental monitoring applications. These applications essentially require the challenging combination of large-scale and low-cost sensor networks that can operate reliably and autonomously over extended periods of time. Still, there is a significant gap between the current state of the art in both in-situ wireless sensor networks and analytical instruments, and what is needed to realise this vision. The marine environment is a harsh environment for sustaining in-situ instrumentation, and in times of extreme events like flooding or storms such instrumentation is prone to failure. Sophisticated analytical instrumentation such as biological or chemical sensors have issues of both reliability and cost [2]. This is because they usually have a limited lifetime in terms of the number of readings they can take before requiring maintenance or re-calibration.

In our work reported here we address both *scalability* and *reliability*. We propose the coordination of multiple heterogeneous information sources to allow more efficient performance of the more sophisticated in-situ analytical instruments in the network. This can extend their lifespan and render them more cost-efficient whilst reducing maintenance requirements because they can adaptively change their operation depending on events at the site. Multiple heterogeneous information sources can characterise the site from multiple different perspectives and thus provide an optimum view of events. If there are a limited number of nodes available for deployment in the network this may also help to inform the placement of these sensors so that they are deployed at the most suitable locations.

Our vision of future large-scale deployments of chemical and biological sensor platforms is based on low-cost somewhat unreliable devices that will modify the operating characteristics of the more sophisticated platforms that are less densely distributed [4]. This presents a grand challenge in research which are for example addressed by the Future Internet objective of the European Commission's Seventh Framework Programme (FP7) [5]. The principle of operation here is that sensors can be used collaboratively in an hierarchical network with the more sophisticated sensors in the network providing highly reliable validation measurements with their duty cycle linked to information provided from less sophisticated nodes. This reduces the duty cycle of the more sophisticated and expensive nodes and increases the overall efficiency of the sensor network [2]. Group behaviour strategies may be used to identify irregular signals and device malfunction and may provide information about the source, dynamics, trajectory and area of effect of an event [6], though the whole issue of managing and accounting for the data quality of marine sensors remains a challenge. In recent work by Timms *et al.* [7], a framework is presented to automatically assess data quality from marine sensor data, which effectively allows the error bars associated with sensor readings to be incorporated into decision-making based on such data, all based on Fuzzy Logic [8]. This information can also be used in deploying the more sophisticated nodes in the network and in related work [9] we describe our own approach to managing data quality but that is beyond the scope of this paper.

In the overall vision of WSNs in environmental monitoring, a community of sensor nodes is based upon a number of sensing modalities. Our particular work focuses on the use of rainfall radar images and information from a water depth sensor in order to dictate the sampling frequency of a a sophisticated phosphate analyser. Other work which we have carried out and reported elsewhere has incorporated camera images and satellite sensors into a network [10–13], similar to the approach advocated by Goddijn-Murphy *et al.* in [14].

In the following section, we present an overview of the study reported in this article. Following this, Section 3 provides an overview of the chemical sensor and the use of rainfall radar information in the context of this research. In Section 4, we describe NNs and their use in hydrological modelling. We subsequently outline our methodology for the application of a NN incorporating rainfall radar information and in-situ depth data for predicting changes in freshwater levels at the Lee Maltings site. In Section 5, we present our results and analysis in relation to the various issues under investigation and finally in Section 6 we present our overall conclusions.

## 2. Study Overview

### 2.1. Method and Objectives

Our study investigates the use of rainfall radar data regularly streamed from the Irish meteorological service web site (http://www.met.ie) and data from an in-situ water depth sensor deployed in a major river for providing contextual information to control the operation of a sophisticated and expensive analytical instrument. More specifically we present a methodology for the incorporation of rainfall radar information from jpeg images and water depth data into a Neural Network (NN) model for predicting average freshwater levels at a river location for controlling the operation of an in-situ phosphate sensor. If a sufficient change in water level is predicted then the phosphate nutrient analyser should be instructed to increase its sampling frequency. During periods where no events of interest are predicted to take place it can remain at a lower sampling frequency. While this may seem a simple example of dynamic adapting, the site we are investigating is complicated because the measurement point is tidal and is downstream of a major electricity generating dam so the river level fluctuates constantly with the upstream tide and downstream water release from the dam. Extracting the true freshwater level with these complications is non-trivial. It also means that we cannot use the high frequency depth data to predict depth values for more frequent intervals than daily intervals. We can only extract freshwater levels when the tide is out which results in 1–2 points per day. However this is sufficient for the purpose in question. Incorporating the rainfall radar information into the model also has the additional benefit of allowing us to examine a number of issues which are outlined below.

Overall this study sets out to determine if we can predict freshwater levels in the River Lee, given overall water depth levels for a number of days past, combined with current and previous rainfall radar information, while taking into account the effects of the tide and the unpredictable release of water from the upstream dam, and varying the combination of lag times of both data sources. However in carrying out this analysis we seek to evaluate a number of additional issues including the following:

- A methodology for incorporation of pixel information from rainfall radar images and in-situ depth data into a NN.
- The most effective way to present rainfall radar information extracted from a digital image—as opposed to raw data values extracted from rain gauges in a catchment area—to the network.
- The effects of rainfall from different points of the catchment area on the model;
- The effect of rainfall and water level information on the model and the effect of differing lag times on the model;
- The accuracy of the NN in predicting freshwater levels.

Our study seeks to demonstrate that with limited training data, a system for controlling the sampling rate of the nutrient sensor can be set up quickly and cost effectively at a deployment and can improve the efficiency of the more sophisticated nodes of the sensor network. The novelty of this work lies in the successful combination of observations from multiple sources for optimization of an environmental monitoring network. The investigation of some of the additional issues outlined above may be very interesting for future flood prediction systems. This work forms part of a broader research effort which is incorporating a number of sensing modalities including cameras, satellite imagers and contextual data into an environmental monitoring network. This leads to increased information, improved detection of marine events, more effective decision making and the more efficient use of sensing technologies in the network. The following section describes the analytical sensor used in our study and how it would benefit from smarter, more adaptive sampling.

### 2.2. Case Study

**Figure 1.**
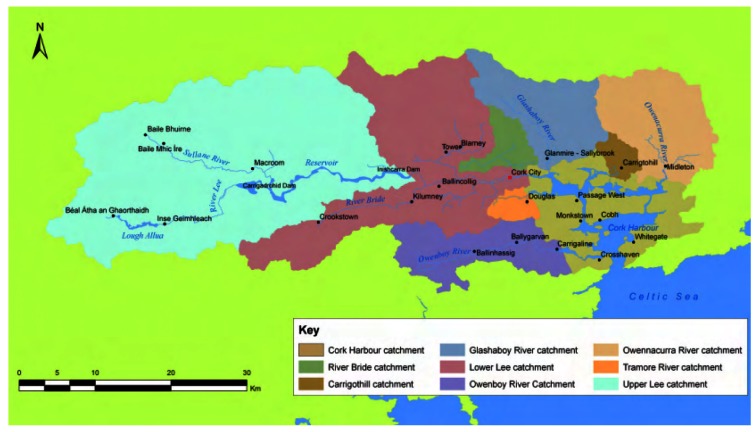
The nine sub-catchment areas of the Lee catchment. For the purposes of this study we are only interested in the Upper Lee and the Lower Lee sub-catchment areas, as the catchment areas designated the 'Lee catchment' encompasses catchment areas of other rivers also [15]. (c) Office of Public Works, 2008, reproduced with permission.

We illustrate our work with a case study carried out on the River Lee, Cork, Ireland. The River Lee represents one of the largest rivers in the Southwest of Ireland. It and its main tributaries drain a catchment area of approximately 1,200 km^2^ upstream of Cork City [15], as shown in Figure 1. The site chosen for our study is the point of the River Lee near where it flows into the sea commonly referred to as the Lee Maltings. This site was chosen due to the deployment of a range of in-situ sensors monitoring a variety of parameters and it is also quite interesting from an environmental monitoring perspective since there are a number of dynamics in place at the site. Water levels at the site are influenced by spillage from the Iniscarra dam and the site is also tidal with a tidal range of approximately 4 metres. The site is instrumented with other sensors for carrying out other aspects of our research including two cameras, one of which has been used for estimating water levels [11,12]. It is also historically important as it was used by the mathematician George Boole in his book “An Investigation of the Laws of Thought on which are Founded the Mathematical Theories of Logic and Probabilities”, published in 1853, as a worked example to illustrate how to combine probabilities of independent events [16].

For this study, the areas delineated as the Upper Lee and the Lower Lee in [15] were used for selecting the catchment area from which to extract the rainfall radar information. In [15] the Upper Lee catchment is said to encompass an area of 790 km^2^. In this area, the peat uplands and steep topography give an elevated runoff potential. In [15], the sub-catchment area outlined as the Lower Lee extends downstream of the Inishcarra dam to Cork Harbour over an area of approximately 420 km^2^ and has a lower runoff potential than the upper Lee catchment. The River Lee flows primarily in an East-West direction from downstream of the Inishcarra dam through Cork City where it then discharges into Cork Harbour. The tidal cycle in Cork Harbour greatly influences water levels of the river in Cork City.

DEPLOY (http://www.deploy.ie) [17], is a technology demonstration project showing an implementation of state of the art technology for continuous, real-time monitoring of a river catchment. The project began collecting data from five sites on the River Lee at 10–15 min intervals from April 2009 until May 2010. The project was co-funded by the Irish Marine Institute and the Environmental Protection Agency (EPA) and was seen as a step towards the realisation of a wide area network of autonomous sensors for monitoring the temporal and spatial distribution of various water quality and environmental parameters.

**Figure 2.**
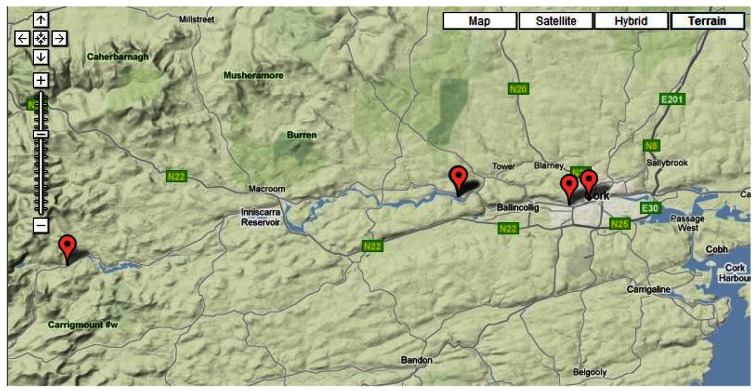
DEPLOY sites on the river Lee. Source: Google Maps and www.deploy.ie.

The monitoring sites for DEPLOY are located in four zones representative of varying conditions along the river and shown in Figure 2. One station is near the source of the river at Gougane Barra, two stations are in the Inniscarra reservoir, one station is in the main channel of the river (Lee road) and the final station, is the site incorporated into this study and it is located in Cork City *i.e.*, Lee Maltings. These zones are considered typical of significant river systems, with stations situated at the source, reservoir, main channel, and an estuary [18].

The Lee Maltings site is located on the north channel of the river Lee at the Tyndall National Institute near the upper end of the estuary on a left hand bend of approximately 70° [19]. It is tidal and partially saline and during the summer months large sections of the river bed tend to dry, however from October this rarely happens. Instruments deployed at this site include off-the-shelf commercial sensors for monitoring conductivity, chlorophyll-a-fluorescence, dissolved oxygen, temperature and water depth. In the context of this study the most important of these sensors is the depth sensor. A phosphate sensor developed in our lab (http://www.clarity-centre.org) can also be deployed at this site. This sensor is a type of chemical sensor previously mentioned that has a limited lifetime in terms of the number of readings it can take before it requires maintenance, hence if samples could be taken more intelligently, it would greatly improve the usefulness of the sensor and extend its lifetime. In the following section we describe these other sensing sources incorporated as part of this study—the phosphate analyser and also the rainfall radar images.

## 3. Sensors Involved

Along with the depth sensor outlined above from the DEPLOY project, this study is working with a sensor for monitoring phosphate and rainfall radar images as a contextual data source. The following provides an overview of these two sensing modalities.

### 3.1. Chemo-Biosensing in Marine or Freshwater Environments

Environmental monitoring applications generally require a high rate of both spatial and temporal sampling. Analytical instruments thus need to be small, portable, environmentally compatible, robust, inexpensive to own and operate, and capable of providing reliable analytical information over extended periods of autonomous operation [20]. Diamond [6] layers analytical devices into a hierarchy in terms of sophistication, capabilities, operational costs and degree of autonomy, outlining a significant correlation between these factors and density of distribution. The key challenge is outlined as driving devices towards the more densely distributed layers by lowering cost while maintaining their reliability and data quality. The most densely distributed layer is dominated by the use of physical transducers, such as pressure and temperature sensors. While transducer-based WSNs are important, it is the introduction of chemo-biosensing that will really lead to greater understanding of environmental processes.

However, the current state of the art in this technology is not ready for large scale deployments.

### 3.2. Autonomous Chemo-Biosensing with Potential for Scale-Up

Although we are still considerably far away from low cost, reliable, self-sustaining chemo/bio sensing devices, there are suggestions for research that could have a revolutionary impact and strategies that offers routes to making progress in the medium term [2,4,6,21]. Many of these interim solutions would benefit from a smarter more adaptive sensor network, operating with an awareness of the environment and changes to that environment. However this needs to occur without significantly increasing the complexity or the cost of the device.

A possible medium-term solution for chemo-biosensing capable of an intermediate degree of scale-up is the use of microfluidics and lab-on-a-chip technology as used in the phosphate analyser on which this research is based. According to Diamond *et al.* [2], the concept of “micro-total analysis systems” or *μ*TAS was introduced by Manz *et al.* [22] around 1990 and became known as lab-on-a-chip (LOAC). In principle, LOAC devices offer a route to incorporation of sophisticated chemo-bio processing in a compact, low-power platform [6]. They offer a compromise between existing lab-based instruments and completely self-sustaining miniaturized sensors and are capable of scale-up. As outlined by Diamond *et al.* [4], the key component of such a device from an analytical perspective is the microfluidic manifold through which samples are accessed, reagents are added, measurements are made, and calibration is performed [23]. In its ultimate manifestation, this concept provides a route to the generation of field-deployable micro-dimensioned analytical instruments that could operate autonomously over relatively long periods of time.

One of the downsides with LOAC devices is that they can store only a limited amount of reagent and waste and they generally require a lot of power for their operation [2]. Thus they can only take a limited number of readings before maintenance is required. If these readings could be scheduled more effectively it would improve the efficiency of the device in terms of reagent consumption, power, maintenance, *etc.* as well as improving the deployment lifetime of the instrument. Such a device has been developed and deployed by our colleagues for monitoring phosphate levels in water (lakes, river, wastewater treatment plant outlets *etc*.) [24]. Its portability, small size and potential low cost renders it very promising for some degree of scale-up in the marine environment. Our work seeks to improve the efficiency of such a device by controlling the sampling rate based on contextual information from other sensing modalities in the environment such as rainfall radar and water depth sensors. The sampling rate can be controlled using the output of the NN which incorporates data from these sensing modalities and predicts freshwater levels. If there is an increase then the sampling rate may need to be changed in order to capture the dynamics and possible nutrient loading in an oncoming event. Otherwise the sampling rate can remain quite low, which improves the efficiency of the sensor. In the following section we describe the site chosen for our study and the in-situ sensor network for real-time monitoring deployed at the site. In the following section, we describe the processing of the rainfall radar images and the extraction of the rainfall data from a catchment.

### 3.3. Rainfall Radar for Calculating Catchment Area Rainfall

In meteorological and hydrological analysis, rainfall radar is a useful measure of precipitation for applications which need to estimate rainfall over a wide area. It is thus very attractive for short term rainfall prediction, called *nowcasting* over a large area with high precision and short lead time [25]. For example the system described in [25] has a maximum lead-time of one hour. Using rainfall radar is different to using traditional numerical weather prediction (NWP) methodologies because it can capture the real time distribution of precipitation, while the NWP models usually have a longer lead time. Rainfall radar analysis thus shows significant advantages when a reduced time factor is important in such applications as flood prediction and event planning.

Met Éireann is the Irish meteorological service and it uses two rainfall radar stations, at Dublin and Shannon respectively, whose images are then merged together to form one overall radar image of the whole country. The images are then published on the Met Éireann website. The radars scan every 15 min to a range of 240 km with a 1 km resolution. The rainfall radar image is converted from reflectivity data in the form of a volume scan which is a sequence of sweeps for increasing antenna elevation angles. The reflectivity is collected on a polar grid with a resolution of 1 km × 1 km.

**Figure 3.**
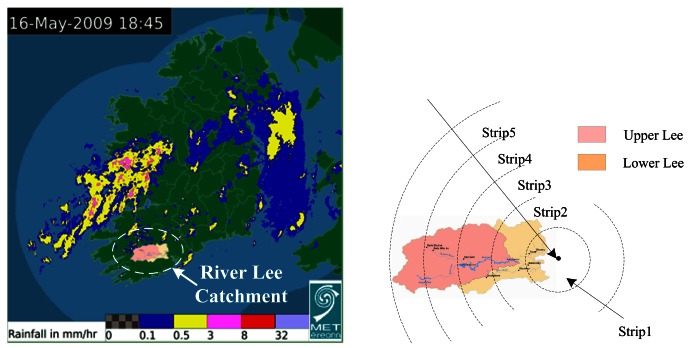
Catchment range for River Lee (left) & strips distribution (right).

Rainfall radar images which show the precipitation distribution and dynamic development over a large area are useful sources of information for estimating overall precipitation in a river catchment area. In this paper, the rainfall within the catchment area of the River Lee is extracted by processing radar images. The catchment contour for the River Lee is illustrated in Figure 3. The catchment area was divided into five strips, each increasingly distant from the point where the river flows into the sea, and the correlation of rainfall from different strips with the water levels in the River Lee, as shown in Figure 3, was calculated. Due to the lag time between rain falling in one of the catchment strips and it affecting the river water level downstream, the strips at various distances from the city area not only reflect the spatial relationship but also reflect the temporal characteristics of the hydrological responses induced by rainfall, or in other words, the delay between rain falling and the freshwater level rising at the city end of the river.

**Figure 4.**
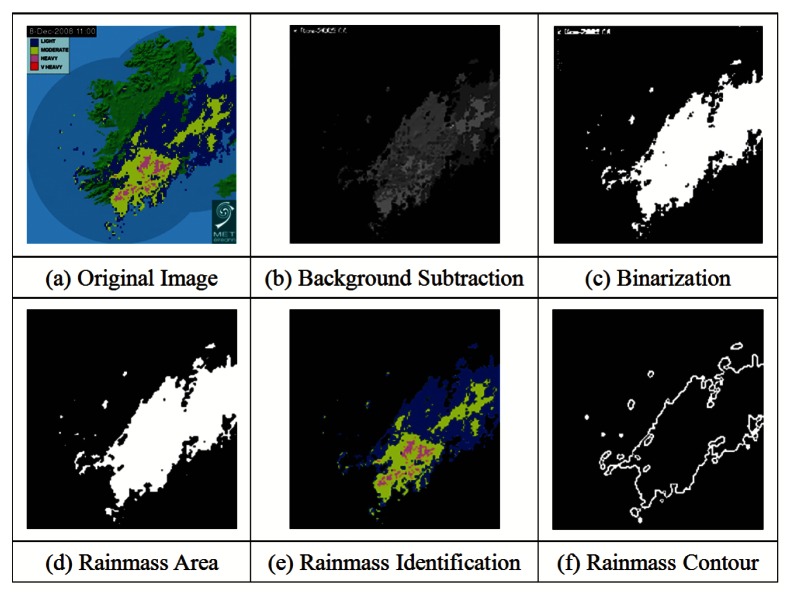
Rainfall Radar Image Processing.

Rainfall radar image processing is employed after each real-time rainfall radar image is collected from the Met Éireann website and features such as rainfall area and intensity are identified. In the radar map, the base map of the country is overlaid with coloured pixels indicating different levels of rainfall intensity from blue to yellow, to pink, to red. Pixels congregate into rainclouds which are clearly visible, as shown in Figure 4. To identify rainfall and its corresponding distribution (combining both intensity and duration) in each catchment strip, we apply a 4-stage process.

1. **Background Subtraction:** Rainmass areas are detected in the original image (See Figure 4(a)) and background subtraction removes the non-rainmass area from the source image (See Figure 4(b)). The image areas representing the rainmass are left with a black background.
2. **Non-Interest Reduction:** Binarization is first applied so that small pixel clusters and non-interest regions can be removed to help with the identification of clusters corresponding to rain showers. These small clusters correspond to reflection from mountains, interference from WiFi or very small and localised rain showers which would not affect river levels as the rainfall would evaporate. This step combines the use of low-pass filtering and macro-block expansion analysis (See Figure 4(c).
3. **Rainmass Identification:** The rainmass areas can be identified as coloured regions, as shown in Figures 4(d,e). Each rainmass contains different intensities of rainfall which are used in data extraction, such as the identification of centre of gravity and intensity of rain.
4. **Edge Detection:** The contour of each rainmass is recognised using an edge detection algorithm to analyze the overall shape of the rainmass. The outputs of the process are black and white images where white pixels represent the contour of the rainmasses (See Figure 4(f)). With the edge detected for rainmass, the rainfall distribution in each catchment strip in Figure 3 can be determined.

The extracted rainfall distribution features are used in the NN-based algorithm for further prediction, which is described in the following section.

## 4. Incorporating Depth and Rainfall Radar into a NN for Predicting Water Levels

Here we investigate the use of a NN for predicting fresh water levels at the site for a given day. The NN incorporates information from the DEPLOY water depth sensor and rainfall data extracted from rainfall radar data provided by the Irish meteorological service. NN's have been widely used in the literature for modelling various non-linear hydrological processes e.g., [26–28]. They have been demonstrated to outperform traditional statistical models and produce comparable results to conceptual models e.g., [26]. Comprehensive reviews on the application of NNs to hydrology can be found in Govindaraju and Rao [29] and Maier and Dandy [30].

- The most effective way to present rainfall radar information extracted from a digital image—as opposed to raw data values extracted from rain gauges in a catchment—to the network.
- The effects of rainfall from different points of catchment on the model;
- The effect of rainfall and water level information on the model and the effect of differing lag times on the model;
- The accuracy of the NN in predicting freshwater levels.

As previously outlined, the chemical sensor we use is a phosphate nutrient analyser [24]. If there is heavy rainfall and run-off from further up in the river catchment area leading to a subsequent increase in freshwater level at the Lee Maltings, then this type of sensor should increase its sampling frequency. Rainfall and subsequent run-off may indicate the influx of nutrients into the water especially if the catchment area consists of land mainly used for pasture grazing or cultivation [31,32]. Thus if an increase in fresh water level can be predicted then the phosphate sensor should be instructed to increase its sampling frequency in anticipation of a possible pollution event. The specific threshold of change that would notably affect phosphate levels cannot be pre-specified, since this would be dependent on a variety of factors. However after field trials, it is hoped a greater indication would be provided. However during periods with little likelihood of phosphate pollution events, the sensor should remain at a lower sampling frequency.

### 4.1. Neural Networks

A Neural Network (NN) [33] is a mathematical model that consists of a network of interconnected elements known as neurons. Signals are presented to the NN through input units which are then propagated and transformed through the network towards the output neurons(s). Each neuron has a number of input arcs (coming from other neurons or from outside the network) and a number of output arcs. The output of a neuron is based on the weighted sum of all its inputs, that is then transformed by an activation function. The output of a neuron is then propagated to subsequent neurons, and onwards. Depending on the type of network and training algorithm employed, the activation function may be logistic sigmoid, linear threshold, Gaussian or hyperbolic tangent functions, and can introduce nonlinear behaviour to the network. Most studies use the logistic sigmoid or hyperbolic tangent functions [34–36]. Works such as by Bishop [37] or Haykin [33] provide detailed discussions on network types and training algorithms.

**Figure 5.**
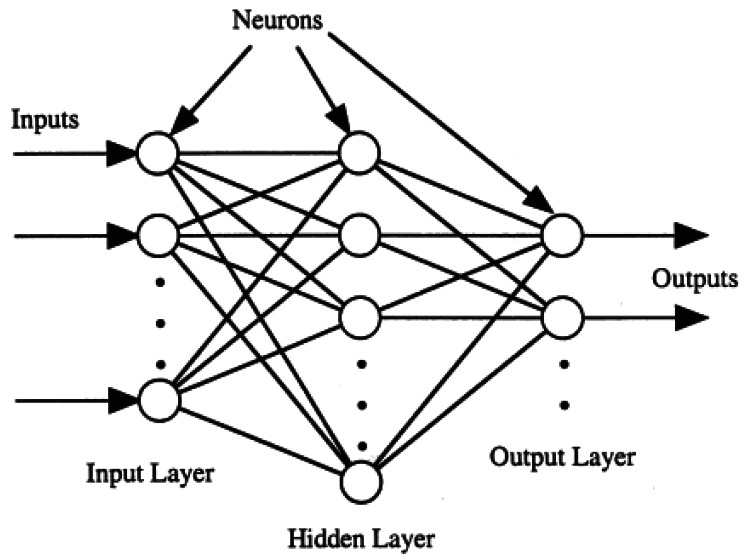
Image: [34] The structure of a feed-forward neural network.

In feed-forward NNs, connections flow in one direction between neurons from the input layer, through one or more hidden layers, to an output layer (see Figure 5). There are many issues that need to be considered and a number of decisions that need to be made in applying NNs to a problem such as ours. In the following section we provide a brief overview of NNs in hydrological modelling applications.

### 4.2. Neural Networks in Hydrological Modelling

One of the main research challenges in hydrology is the development of computational models that are able to accurately simulate the response of a catchment to rainfall. These computational models are categorised according to the approach used and de Vos and Rientjes [35] outline two main categories—knowledge-driven approaches and data-driven approaches. Techniques involved in data-driven modelling are outlined as mainly originating from the field of statistics and artificial intelligence (e.g., time series, empirical regression, fuzzy rule-based systems and NN modelling), where as knowledge-driven modelling aims to reproduce the real-world hydrological system along with its behaviour in a physically realistic manner. The drawbacks with physically-based models are that they have excessive data requirements, over-parameterisation effects, parameter redundancy effects and large computational demands. Instead, data-driven approaches do not suffer many of the disadvantages associated with knowledge-driven models, however they do have other issues. For example, the range of applications may be limited due to the fact that they are developed from a set of records used for model calibration and thus they may not extrapolate well into future situations.

The use of NNs has gained significant attention from hydrologists in recent years for modelling water level patterns in a river system. Comprehensive reviews of the application of NNs to hydrology have been carried out, outlining a framework for the development of NN prediction models in hydrology and a description of the various considerations in their application e.g., [30,34]. Many authors have highlighted their benefits in such applications, e.g., [26,27,34,38,39]. NNs do not pre-suppose a detailed understanding of a catchment's physical characteristics or require extensive data preprocessing, and they are also noted to be quite effective for handling incomplete, noisy and ambiguous data. Zealand *et al.* [40] highlights their capability for constructing complicated non-linear models for multivariate time-series. They also note issues in relation to the statistical distribution and stationarity of the data. To optimally fit an AutoRegressive-Moving-Average (ARMA) type model to a time-series, the data must be stationary and follow a normal distribution. Instead, when developing NN models, the statistical distribution of the data does not have to be known and the internal structure of the NNs implicitly account for non-stationarities in the data, such as trends and seasonal variations. Good generalization capability is also outlined as an advantage of NNs as unlike ARMA-type models they are relatively insensitive to noisy data and they they have the ability to determine the underlying relationship between model inputs and outputs.

In the literature, NNs have been demonstrated as a tool capable of modelling various non-linear hydrological processes. Coulibaly *et al.* [28] points to studies which have demonstrated that they offer a promising alternative for rainfall-runoff modelling, streamflow prediction, and reservoir in-flow forecasting. They have been demonstrated to outperform traditional statistical models and produce comparable results to conceptual models, e.g., [26]. Comprehensive reviews on the application of NNs to hydrology can be found in Govindaraju and Rao [29] and Maier and Dandy [30]. In the following we outline our methodology for the application of NNs for predicting freshwater levels at the Lee Maltings site which is adapted from the framework outlined in [34].

### 4.3. Using Neural Networks for Predicting Freshwater Levels at the Lee Maltings

**Figure 6.**
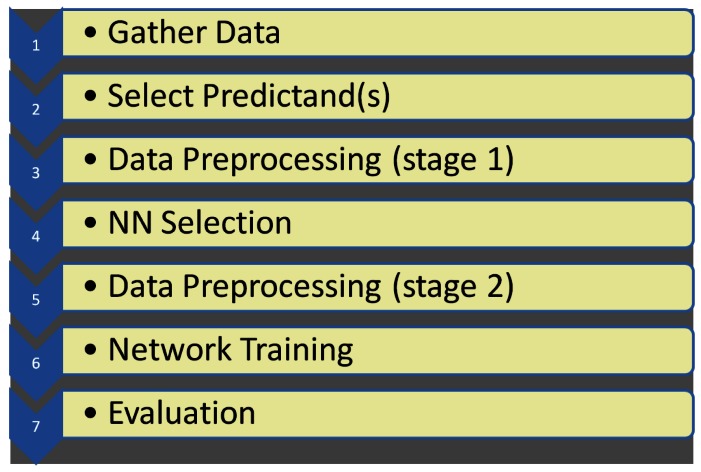
Framework outlined by Dawson and Wilby [34] for the application of NNs to rainfall-runoff modelling and flood forecasting, which will be applied in the context of our study.

In contrast with other studies in the literature which investigate the use of NNs in hydrological modelling, our objective is not to predict water levels or water flow at the site in question, but to predict average freshwater level at a site which is influenced by the tide and a hydroelectric dam further upstream. Predicting average freshwater level for the current day is considered sufficient as it allows the operation of the sensor to be alerted in sufficient time to modify its operating characteristics in order to capture the dynamics of any impending event. The methodology we applied is adapted from the framework outlined by Dawson and Wilby [34] for the application of NNs to rainfall-runoff modelling and flood forecasting. This framework consists of seven stages and our methodology will be described under the headings outlined in Figure 6.

#### Step 1. Gather Data

This involves gathering data for the training and testing of the model. Depth data for the Lee Maltings site was gathered from a water depth sensor deployed as part of the DEPLOY project at a sampling rate of up to 144 samples per day. Rainfall data was provided by rainfall radar images from the Met Éireann website updated every 15 min resulting in 96 images per day. For each of these sensor streams there are some gaps in the data due to issues with the sensor such as biofouling, or issues with the network such as network downtime.

#### Step 2. Select predictand(s)

In applying the NN, the model application needs to be defined and outlined. In the context of this study, the model application is the prediction of average freshwater level at the site for the current day. As previously explained in Section 2 the site is tidal, which means that we cannot use the high frequency depth data to predict depth values for more frequent intervals than daily intervals.

#### Step 3. Data preprocessing (stage 1)

Dawson and Wilby [34] outline two steps involved in the data pre-processing stage—data cleansing and the selection of inputs/outputs or predictors/predictands however some authors have suggested that extensive data pre-processing does not have to be considered when employing NNs, e.g., [40] and that it is not considered by many studies, e.g., [30].

We found the most reliable approach to extracting freshwater levels for pre-processing of depth data at the site is to extract the minimum water level from each tidal cycle. This was following an approach whereby we used conductivity data to determine freshwater levels. However we found the conductivity data was not always reliable e.g., at periods where the sensor required maintenance, and was sometimes indicating incorrect levels. Thus for each tidal period the minimum point of that period is extracted as an input to the NN model. This resulted in approximately two water depths per day since there are generally two tidal cycles occurring within a 24-hour period.

In the rainfall radar images from the Met Éireann website, the map of Ireland is overlaid with coloured pixels indicating five different levels of rainfall intensity namely very light, light, moderate, heavy and very heavy. The number and location of each of these types of rainfall/pixel colours are extracted for each of the five strips of the catchment. This produces a dataset with five data points for each of the five strips of the catchment for each image. Each data point represents the area of the catchment (in km^2^) subject to the type of rainfall in question in that image.

The rainfall radar dataset is aligned with the water depth dataset for creating a set of instances for input into a NN. Because of occasional gaps in the rainfall radar data, and the differing sampling rates between rainfall (one every 15 min) and freshwater level readings (1–2 per day), we calculated an average freshwater level and and average rainfall value for each day. Two main categories of datasets were produced:

- For each day, *the average fresh water level* + *the average of the catchment coverage of each of the 5 rainfall intensity levels in km*^2^ (*i.e.*, values in km^2^ are collected every 15 min for each rainfall type, hence the daily average for each rainfall type is produced resulting in 5 values—one average for each intensity level) + *the overall average catchment coverage of rainfall in km*^2^ (average of the 5 averages produced for each intensity level), per catchment strip;
- For each day, the *average fresh water level* + *rainfall in mm*, per catchment strip.

#### Step 4. Neural Network Selection

In this part of the methodology, Dawson and Wilby [34] outline two tasks—the selection of a network type and a training algorithm. textcolorredIn the literature two types of feed-forward network are often used in modelling processes similar to rainfall-runoff: the multilayer perceptron (MLP) and the radial basis function network (RBFN) [34]. The network type chosen for our work is the MLP which comes as part of the WEKA data mining software toolkit (http://www.cs.waikato.ac.nz/ml/weka/) [41]. This is one of the most popular network types used and is trained using the error backpropagation algorithm. Sigmoidal type functions such as the logistic and hyberbolic tangent functions are the most commonly used transfer functions [30] and in our work a sigmoid activation function is used. A number of parameters affect the performance of the training algorithm including step size [30]. Generally a trial and error approach is used in order to optimise this and it is normally a function of a number of network parameters such as learning rate, momentum, error function, epoch size and the gain of the transfer function [42]. Dawson and Wilby [34] mention choosing appropriate values for momentum and learning rate within the range 0.01 and 0.9. Hence we optimised these two parameters and otherwise used the default parameters in the WEKA toolkit. Following optimisation, we chose a learning rate of 0.1 and a momentum rate of 0.1 as the network appeared to become quite unstable for higher values.

#### Step 5. Data preprocessing (stage 2)

Firstly this part of the methodology involves data standardization or normalization. Dawson and Wilby [34] state that in general, data are rescaled to the intervals (−1,1), (0.1, 0.9) or (0,1). The WEKA toolkit automatically normalises data within the range (−1,1). The next step is to split the data into training sets and test sets. Training data ranges from 15 May 2009 until 31 January 2010. However due to gaps in datasets, there is a limited number of training instances once the data is aligned and a consecutive number of days of each data source is required for one instance. The training dataset is composed of 129 instances with instances available from the months May (from 15 May onwards), June, September, December, January and more limited instances then available for July, August, October, November. With limited data availability, a cross-training technique is often adopted [34]. Ten-fold cross validation is a standard technique used in in the machine learning literature for evaluation of models and this is the technique employed in this study. A set of data was held out in which to test the final model. This data ranged from 1 February to 4 June 2009. There were less gaps in the data during this time period and it resulted in 118 test instances.

#### Step 6. Network Training

This part of the methodology involves specifying the number of hidden layers and the number of nodes in these layers. There exist various approaches for determining an appropriate number of hidden nodes in the network. However some authors believe the best approach to be via trial and error, e.g., [43]. We adopt a trial and error approach whereby hidden nodes from 2 to 50 in steps of 2 are examined using one hidden layer. This range was chosen since initial evaluations demonstrated that over 50 hidden nodes resulted in quite a slow network producing lower correlations. Following the examination of results, we choose to report the results for networks with 2, 12, 22, 32, 42, and 50 hidden node values for each model evaluation. These values can have a large effect on model output.

#### Step 7. Evaluation

Finally in the last stage of the framework, the output of the NN needs to evaluated using appropriate error measures. For evaluation of the best-performing models on test data both correlation with observed depth and mean absolute error (MAE) values will be reported. These measurement values are appropriate for the purposes of this study.

## 5. Results and Discussion

Our measure of performance is to correlate the predicted level against the observed levels for the following day. A number of different input models to our NN were examined which vary in terms of:

- **rainfall information**—presented as individual rainfall classes or as an aggregated values across all rainfall classes.
- **lag times**—different combinations of lag times were examined with up to 5 days of prior rainfall information presented to the NN (current day and 4 antecedent days).
- **catchment strips**—we varied inputs in terms of information from one catchment strip or a combination of information from all catchment strips.

We defined 6 different models as combinations of these inputs, shown in Table 1. The first two of these are used where networks are developed separately for each strip of the catchment. This is to determine the effect that rainfall information from each strip of the catchment has on the NN performance and to see whether information from a particular part of the catchment has more influence on prediction performance than other parts.

We carried out a large number of experiments on data from the River Lee, varying all input parameters individually and in combination but for conciseness we present only the most interesting of the results. In particular we examine two research questions, namely the effect of rainfall from different areas of the catchment on prediction performance, and the most effective way to aggregate prior rainfall for maximising prediction accuracy. Following this we determine the parameter combinations which maximise performance of the NN for predicting freshwater levels and we discuss the reasons for these parameter combinations, evaluating the overall performance of the network at accurately predicting freshwater levels. The correlations described below are based on 129 data points, apart from at the end of this section where the final models are evaluated on test data which consists overall of 118 data points.

### 5.1. Effects of Rainfall from Different Parts of the Catchment on Prediction Performance

In order to examine if rainfall from specific parts of the catchment have more of an effect on prediction accuracy than others, we compare the correlations output by the NN with a varying number of days of rainfall and water level values for each strip of the catchment. We examined the output for models with between one and five days of rainfall information and zero or two preceding days of water depth values. This allows us to examine the effect of rainfall from each catchment strip, with and without the additional water level information, on the NN.

**Figure 7.**
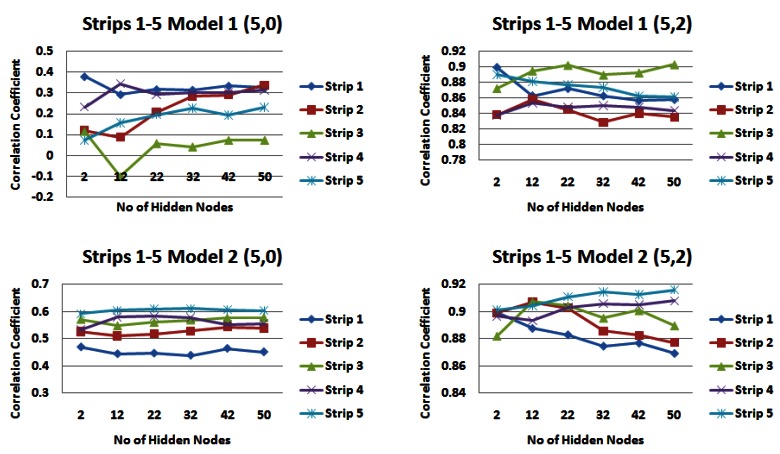
Correlations for input models 1 and 2 for 1 day rainfall information and 0 and 2 days water level information for each strip of the catchment.

**Figure 8.**
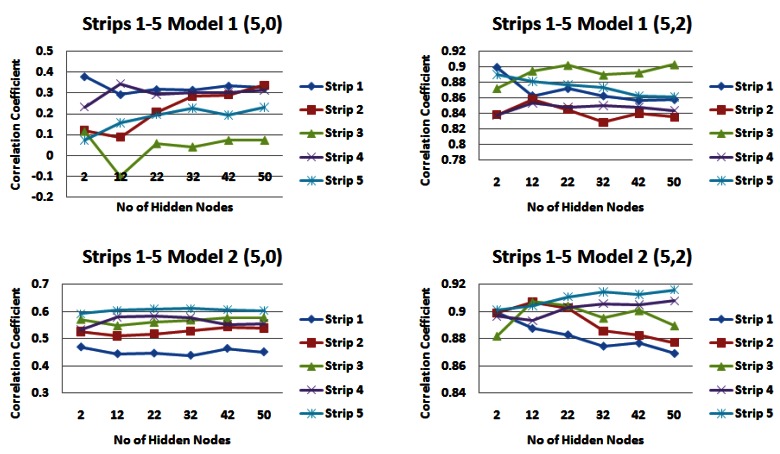
Correlations for input models 1 and 2 for 5 days rainfall information and 0 and 2 days water level information for each strip of the catchment.

Tables 2 and 3 highlight the catchment strips that appear to produce the highest correlations for each of the input models across different combinations of the number of rainfall and water level values. For demonstration purposes, Figure 7 shows the results for input models 1 and 2 consisting of 1 day of rainfall information and two different values of water level information (zero or two), Figure 8 shows the same for input models 1 and 2 with five prior days of rainfall information.

From the results of this part of the analysis it is clear that **strip 4** appears to be a very dominating strip of the catchment featuring heavily in the results of both input models. This is a very exciting and interesting outcome considering the description of the upper Lee Catchment in Section 2.2 on page 4609 outlines a slightly elevated runoff potential due to the peat uplands and steep topography. Overall it is *input model 2* that generally produces the higher correlation values.

### 5.2. Presenting Rainfall Radar Information to the NN

Here we investigate the most effective method to present rainfall radar information to the NN through examining the correlations output by the various input models. As previously outlined these differ in the manner in which this data is presented to the network. We examine the outputs of the network for combinations of 1, 3, and 5 days of rainfall information and 0 and 2 days of water level information.

#### 5.2.1. Input Models 1–2—Strip 4 of the Catchment

**Figure 9.**
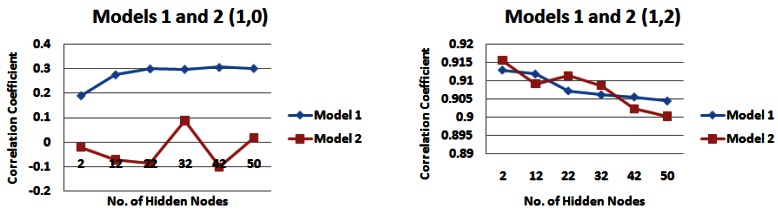
Correlation coefficients for input models 1 and 2 for 1 day rainfall information and 0 and 2 days water level information for strip 4 of the catchment—*(**a**) Models 1 and 2 (1,0) (**b**) Models 1 and 2 (1,2)*.

**Figure 10.**
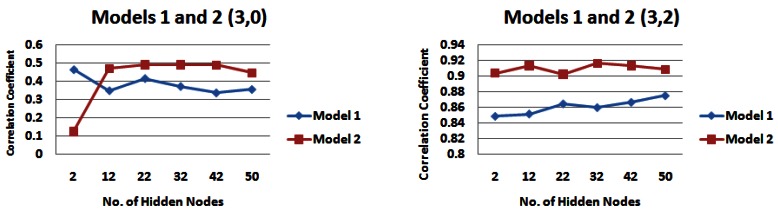
Correlation coefficients for input models 1 and 2 for 3 days rainfall information and 0 and 2 days water level information for strip 4 of the catchment—*(**a**) Models 1 and 2 (3,0) (**b**) Models 1 and 2 (3,2)*.

**Figure 11.**
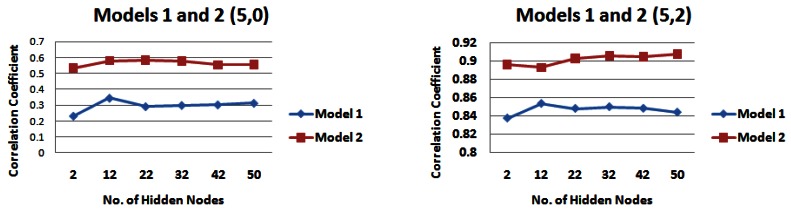
Correlation coefficients for input models 1 and 2 for 5 days rainfall information and 0 and 2 days water level information for strip 4 of the catchment—*(**a**) Models 1 and 2 (5,0) (**b**) Models 1 and 2 (5,2)*.

Since **strip 4** seemed to be a dominant strip of the catchment for a variety of scenarios examined in Section 5.1, we used the results from this part of the catchment for carrying out the analysis here (See Figures 9, 10, and 11). Firstly, we examine the output of the models when no water level information was provided to the network. For 1 day of rainfall information *input model 1* performs best, producing a maximum correlation value of just over 0.30 (Figure 9(a)). However for 3 and 5 days of rainfall information, *input model 2* generally performs best, producing correlation values of just over 0.49 for 3 days of rainfall information (Figure 10(a)) and 0.58 for 5 days of rainfall information (Figure 11(a)). When water level information is added to the model, *input model 2* performs with a higher correlation for 3 and 5 days of rainfall information (Figures 10(b) and 11(b)), with a maximum correlation for 3 days water level information (0.93).

#### 5.2.2. Input Models 3–6—Combination of All Strips

**Figure 12.**
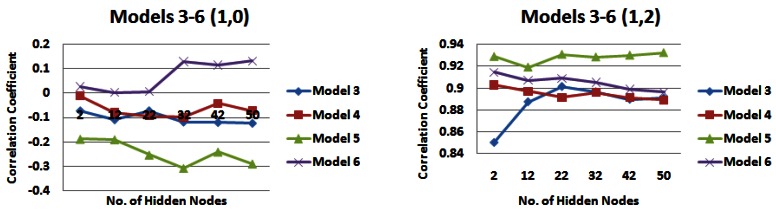
Correlation coefficients for input models 3–6 for 1 day of rainfall information and 0 and 2 days water level information—*(**a**) Models 1 and 2 (1,0) (**b**) Models 1 and 2 (1,2)*.

**Figure 13.**
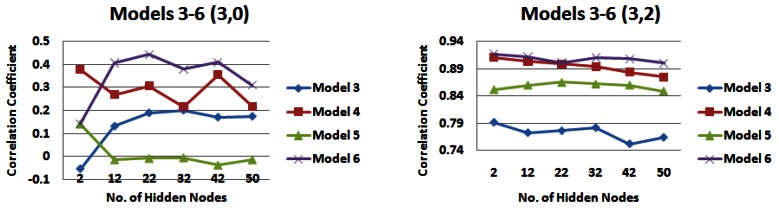
Correlation coefficients for input models 3–6 for 3 days of rainfall information and 0 and 2 days water level information—*(**a**) Models 1 and 2 (3,0) (**b**) Models 1 and 2 (3,2)*.

**Figure 14.**
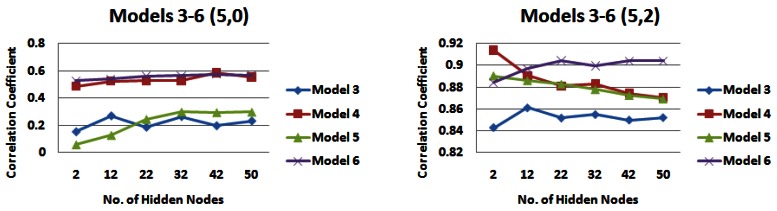
Correlation coefficients for input models 3–6 for 5 days of rainfall information and 0 and 2 days water level information—*(a) Models 1 and 2 (5,0) (b) Models 1 and 2 (5,2)*.

*Input models 3–6*, which incorporated information from all strips of the catchment, were also compared over different combinations of rainfall and water level information (See Figures 12, 13, and 14). When no water level information is input to the network, it appears that *input model 3* generally produces the lowest correlations and *input model 6* generally produces the highest correlations, with *input model 4* also producing similar correlations for 5 days rainfall information (Figures 12(a), 13(a), 14(a)). When water level information is added to the network, *input model 5* outperforms other models for 1 day of rainfall information, producing a correlation coefficient of just over 0.93 (Figure 12(b)). However when 3 or 5 days of rainfall information is added to the network, this model performs poorer than most (Figures 13(b), 14(b)). *Input model 3* is generally the poorest performing overall. For 3 and 5 days of rainfall information, *input model 6* overall generally performs with the highest correlation values, reaching just under 0.92 for 3 days rainfall information (Figure 13(b)) and just under 0.91 for 5 days rainfall information (Figure 14(b)). Similar trends could be seen with 4 days of water level information, but are not outlined here.

From this we can learn that it is clear that applying many rainfall values to the network, where a value for each individual rainfall type for each strip of the catchment is presented to the network, generally results in poorer correlation with the observed depth information. Furthermore, aggregating these values in some manner improves performance.

### 5.3. Performance of the NN for Predicting Average Water Level

From the previous analyses it is apparent that quite high correlations can be achieved with the use of a NN. Based on an analysis of a variety of combinations of lag times, we chose to concentrate on input models with 2 days rainfall information and 2 and 3 days water level information and 3 days rainfall information and 2 and 3 days water level information for examining the overall performance of the NN for predicting average freshwater levels since these configurations seemed to be producing the best performance.

**Figure 15.**
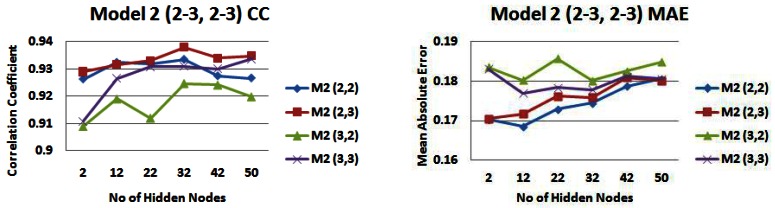
Correlation coefficients and mean absolute error values for model 2 with different combinations of 2 and 3 days rainfall and water level information—*(**a**) Correlation values (**b**) Error values*.

**Figure 16.**
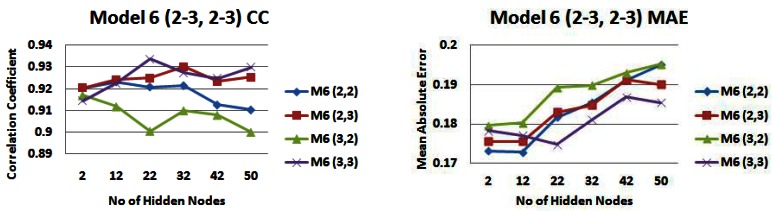
Correlation coefficients and mean absolute error values for model 6 with different combinations of 2 and 3 days rainfall and water level information—*(**a**) Correlation values (**b**) Error values*.

Figures 15 and 16 shows the correlation (R) and mean absolute error (MAE) values of again predicted *versus* actual outputs, for *input models 2* (strip 5) and *6* with combinations of 2 and 3 days rainfall and water level information. For *input model 2*, models with 2 days rainfall information are generally producing the highest correlations with a highest correlation value of 0.938 (2,3) (Figure 15(a)). For *input model 6*, models with 3 days water level information are generally producing the highest correlations with model (3,3) producing the highest correlation value of 0.9337 (Figure 15(a)). For *input model 2*, an input model consisting of 2 days rainfall information and 2 days water level information (2,2) generally produces the lowest MAE, reaching a lowest value of just under 0.17 m (Figure 15(b)). For *input model 6*, apart from small hidden node values, the MAE is generally lowest for an input model consisting of 3 days rainfall information and 3 days water level information (3,3), reaching a lowest MAE of 0.1748 m (Figure 16(b)). Apart from during an event in November where the in-situ sensors went offline, the range of daily average freshwater levels was from 0.35 m to 2.48 m in the training set, hence the performance being produced is very satisfactory, with error values of just under 0.17 m being reached.

### 5.4. Performance on Test Data

**Figure 17.**
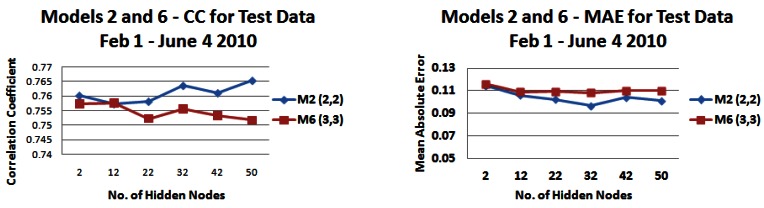
Correlation coefficients and mean absolute errors for input models 2 (2,2) and 6 (3,3) when tested on data from 1 February to 4 June 2009—(a) Correlation Values (**b**) Error Values.

For the final testing of our NN based approach to predict water levels, having varied most of the parameters for NN input and configuration, we use an input model with 2 days rainfall and water level information for input model 2 and 3 days rainfall and water level information for input model 6. These models were tested using data from 1 February 2009 to 4 June 2009, resulting in 118 test instances and the results are shown in Figure 17.

**Figure 18.**
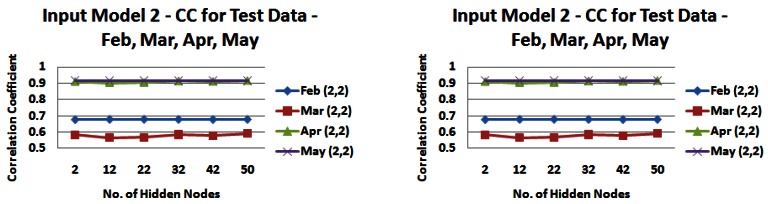
Correlation coefficients and mean absolute errors for input model 2 (2,2) when tested on data from individual months—February, March, April, May—(**a**) Correlation Values (**b**) Error Values.

**Figure 19.**
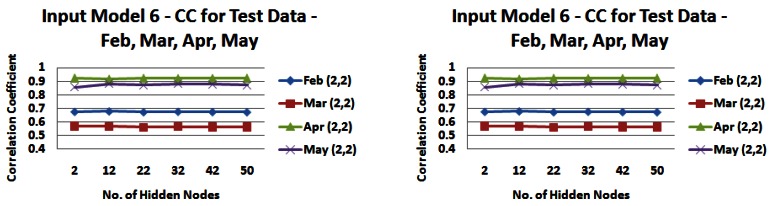
Correlation coefficients and mean absolute errors for input model 6 (3,3) when tested on data from individual months—February, March, April, May—(a) Correlation Values (**b**) Error Values.

It is apparent that the input models perform with a lower CC and MAE on the test data with correlations between 0.7572 and 0.7653 and a MAE between 0.0967 m and 0.1144 m for input model 2, a CC between 0.7517 and 0.7576 and a MAE between 0.1084 m and 0.1161 m for input model 6. The range of daily average freshwater levels was from 0.51 m to 1.89 m in the test set. The range is smaller than that for the training set, which may explain the reduced correlation but lower error values. However overall the error ranges described above are again very satisfactory for the application context in question. In order to investigate the reduced performance in terms of correlation between the actual values and predicted output, we tested the models on a month-by-month basis to see if there was a seasonal difference across the months of February, March, April and May and the results can be seen in Figures 18 and 19).

The graphs in Figures 18 and 19 demonstrate the seasonal effects, with the months of April and May giving consistently good results, with March and to a lesser extent February bringing performance down. It is unclear what is causing this reduction in performance in February and March. It may be due to operations of the dam further upstream whose operation cannot be predicted or accounted for in the model or it may be due to a more limited set of training data for those months. However, despite the reduced performance, the application of the NN for these months is sufficient to predict a change in freshwater levels to instigate a change in the sampling rate of our phosphate sensor and the MAEs produced by this model are sufficient to meet this task. As stated earlier, effecting a change in sampling rate for the phosphate sensor does not require precise measurements of freshwater level.

## 6. Conclusions

We investigated a method for the use of rainfall radar information and in-situ depth measurements to predict average freshwater levels at the Lee Maltings site for controlling the sampling rate of an in-situ phosphate sensor. We investigated a variety of issues, some of which are novel in relation to the use of rainfall radar data as an input data stream to a NN for this purpose. This produced some very interesting results, with the rainfall radar information appearing to determine trends in the catchment and an overall output of the network which is suitable for the application context in question.

Despite the noisy data, it appears that the rainfall radar information determines geographic areas of the catchment corresponding to those implied in the hydrological study of the region [15] that influence runoff and consequent freshwater depth more than others. It can therefore have a positive impact on the network operating as a contextual data source. In terms of the overall performance of the network, our experiments showed varied performance for different times of the year but overall produced output that would be satisfactory for this particular application given that we do not require precise water levels, rather just an estimation. Overall on the test data, *input model 2* produces a MAE between 0.0967 and 0.1144 and *input model 6* produces a MAE between 0.1084 and 0.1161. As previously outlined, the specific threshold of change that would notably affect phosphate levels cannot be pre-specified, since this would be dependent on a variety of factors. However considering the aim is to predict an increase so that the sampling rate can be changed to capture the dynamics of a potential event, this result is very promising.

However, with more training data, it may be worthwhile developing seasonal models and evaluating the issues here further as our analysis overall was carried out with limited training data. This may help to further establish the causes of reduced performance for certain months. In the previous section we could only speculate as to why this reduced performance was occurring. This model is dependant on the training data and it may be the case that the training data does not capture similar trends occurring during these months in the test data. It also be may due to the releases of the dam further upstream which cannot be incorporated into the model. More training data would help to investigate these issues further along with establishing events for which the model can and cannot effectively capture, which may further indicate follow on actions to address these issues.

Overall we have demonstrated that the use of heterogenous sensing modalities as a tool for providing contextual information to control the operation of a more sophisticated analytical instrument in a sensor network. This study demonstrates that even with limited training data, a system can be set up quickly and cost-effectively at a deployment. We have demonstrated an approach that could easily be adapted to any deployment in order to generate a more intelligent overall network that uses its more sophisticated nodes more effectively based on information from other sources in the network. This approach could be further generalised to alternative scenarios. For example this work forms part of a broader research effort which aims to combine data from a multitude of sensor modalities including visual sensing devices. Similarly visual sensing data could be used in a similar manner as a contextual data source to control the operation of a more sophisticated sensor node but also for further scenarios. For example it may be used in order to dispute or validate in-situ sensor readings or to determine events at the site during a time when an in-situ sensor may go offline during extreme events or due to issues with the hardware *etc*. It may also help to determine events that cannot be understood from simply relying on in-situ sensor data from one point of a river. Work has already been carried out in detecting events from the images [13]. The next step is to integrate this as a contextual data source in the network. Techniques involving the use of trust modelling have already been used in order to link this information to the in-situ depth sensor. However further research is required in order to fully investigate how this can be integrated into a multi-modal monitoring network where different modalities can be optimised to their full potential in the network and be integrated to operate alongside and complement the operation of other modalities in the network. It is envisaged that the similar use of a neural network based approach would prove very beneficial in this scenario also.

Using rainfall radar and in-situ depth data to determine changes in freshwater levels may also have additional benefits and uses. If this model can determine parts of the catchment more likely to cause changes in water levels, and can approximate these changes with limited data, then such a tool could be further optimised for flood prediction and for use as a flood management tool. This would involve further analysis on the model outputs. While we have one high water level event in our training data to focus on, we would require a test event in order to evaluate the model's ability to capture and predict such an event. However overall it appears that such an approach has a very promising appeal as a low cost solution with limited data requirements to capturing the dynamics of a river catchment.

## Figures and Tables

**Table 1.** Models developed for water level prediction.

**Input Model**	**Description**	**Strips**
Input model 1	Rainfall information from each rainfall type is presented to the network separately for each day considered *i.e.*, VLIGHT, LIGHT, MODERATE, HEAVY, and VHEAVY.	Developed separately for Strips 1,2,3,4,5
Input model 2	Uses averaged information from all rainfall types so one rainfall value is presented to the network for each day.	Developed separately for Strips 1,2,3,4,5
Input model 3	Similar to input model 1 but with information from each strip of the catchment presented to the network for each day.	Strips 1–5
Input model 4	Similar to input model 2 but with this value presented to the network from each strip of the catchment for each day.	Strips 1–5
Input model 5	Averages the information for each individual rainfall class across all strips for each day.	Strips 1–5
Input model 6	Averages the input values from input model 5 so that one rainfall value is presented to the network for each day.	Strips 1–5

**Table 2.** Strips of the catchment generally (not always) producing the highest correlation coefficients in predicting freshwater levels at the Lee Maltings site where no antecedent water level information is input to the NN model.

**Rainfall Days**	**1**	**2**	**3**	**4**	**5**
**Input Model 1**	4	4	4	4	4,1,2
**Input Model 2**	3,2	4,5	4	3,5,4	5,4,3

**Table 3.** Strips of the catchment generally (not always) producing the highest correlation coefficients in predicting freshwater levels at the Lee Maltings site where 2 days antecedent water level information is input to the NN model.

**Rainfall Days**	**1**	**2**	**3**	**4**	**5**
**Input Model 1**	4,1	4,1	1,4	1,4	3
**Input Model 2**	4,5	5,4	5,4	5,4	5,4
